# Molecular LEGION: incalculably large coverage of chemical space around the NLRP3 target

**DOI:** 10.1038/s41597-026-06850-y

**Published:** 2026-03-03

**Authors:** Bogdan Zagribelnyy, Vladimir Aladinskiy, Nikita Bondarev, Ivan Ilin, Maxim Malkov, Anna Vasileva, Xiaoyu Ding, Arkadii Lin, Rim Shayakhmetov, Alex Aliper, Feng Ren, Alex Zhavoronkov

**Affiliations:** 1 Insilico Medicine AI Limited, Level 6, Unit 08, Block A, IRENA HQ Building, Masdar City, Abu Dhabi UAE; 2 Insilico Medicine Hong Kong Ltd., Unit 310, 3/F, Building 8 W, Phase 2, Hong Kong Science Park, Pak Shek Kok, New Territories, Hong Kong, Hong Kong SAR, China; 3 Insilico Medicine Shanghai Ltd., Suite 901, Tower C, Changtai Plaza, 2889 Jinke Road, Pudong New District, Shanghai, 201203 China; 4 Insilico Medicine Canada Inc., 3710-1250 René-Lévesque Blvd W, Montreal, Quebec H3B 4W8 Canada

**Keywords:** Computational chemistry, Screening, Cheminformatics

## Abstract

The exploration and mapping of chemical space remain a central challenge in modern drug discovery. Traditional compound libraries and databases cover only a minute fraction of this space, limiting the discovery of novel, bioactive, and patentable chemotypes. Here, we present a unique dataset containing approximately 110 M molecular structures of potential NLRP3 inhibitors enabled by the LEGION (*Latent Enumeration, Generation, Integration, Optimization, and Navigation*) workflow, which integrates generative AI, AI-guided screening within the Chemistry42 platform and auxiliary cheminformatics tools to enable large-scale exploration of chemical space around specific drug targets. Using the structural data of NLRP3 co-crystals, a clinically relevant target, LEGION combined ligand- and structure-based design strategies, in-house algorithms for 3D pharmacophore-aware scaffold extraction, and distinct library enumeration methods to identify over 34,000 unique scaffolds, which can be multiplied into a dataset of 123B molecular structures within the provided code. The resulting dataset of unprecedented size proved effective for scaffold hopping, chemical space navigation, and supporting intellectual property applications by generating structurally diverse and synthetically accessible structures.

## Background & Summary

The concept of *chemical space* — the abstract, multidimensional space encompassing all possible small organic molecules — is central to modern drug discovery and cheminformatics. However, this space is astronomically vast, with estimates^1^ up to 10^60^ of only “drug-like” molecules depending on the criteria applied for molecular weight, synthetic accessibility (SA) or/and synthetic feasibility, and physicochemical properties. Enumeration, representation, and exploration of chemical space have become essential tasks for identifying and patenting bioactive compounds.

**Fig. 1:**
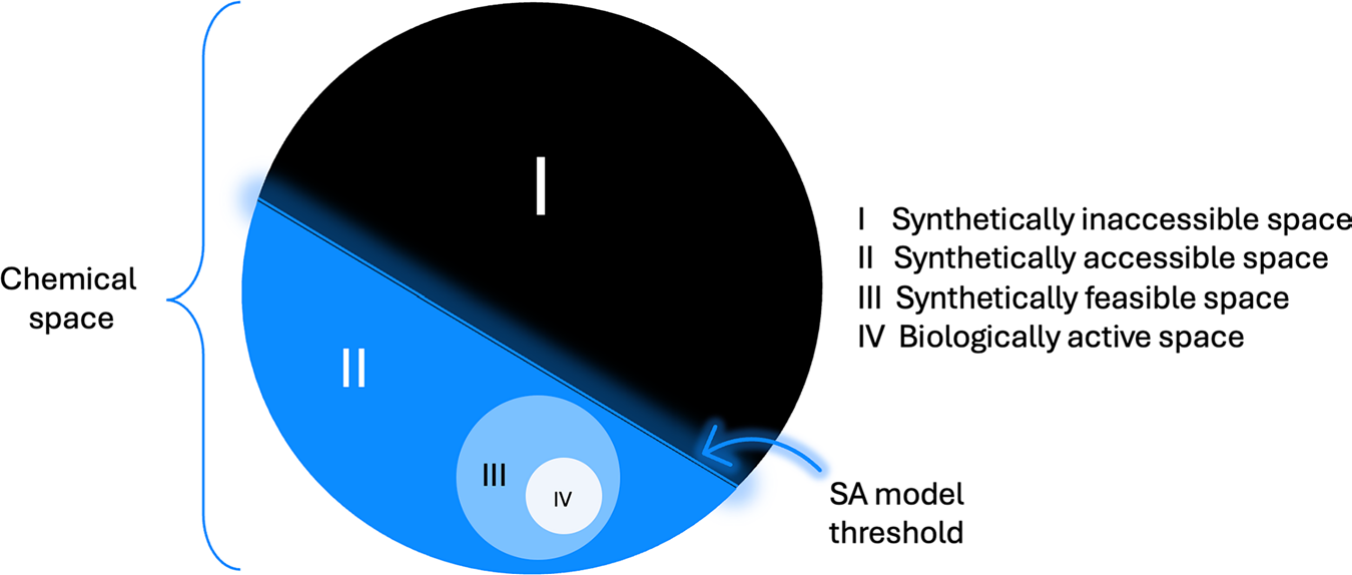
The topology of chemical space.

Despite the availability of large compound databases like PubChem^2^, ChEMBL^3^, and vendor libraries like Enamine^4^ or aggregators like MolPort^5^ — these represent only a tiny fraction of the total accessible chemical universe, the fraction that is actually represented by compounds in immediate availability, mostly synthetically feasible ones (Fig. 1, III)^6^. At the same time, it should be noted that not all biologically active molecules presented in ChEMBL or similar sources are synthetically feasible. These extremely structurally complex molecules are present only in natural sources and have never been synthesized. Here, we should also admit that we are forced to rely on the quality of the named sources, providing mostly synthetically feasible molecular structures, while the artifacts and inconsistencies may occur even in these sources and both manual inspection and quite reliable automatic verification on the feasibility status are impossible on such large-scale datasets. At the same time, an even smaller fraction of the accessible chemical space is formed by biologically active compounds (Fig. 1, IV), while obviously not all of them are drug molecules. The larger fraction at the next level of abstraction is represented by structures that are synthetically accessible, but their synthetic feasibility status is questionable, since no one has synthesized them yet (Fig. 1, II). However, this fraction is the most interesting one, since all novel chemotypes are actually coming from it. Finally, there are a myriad of structures that are valid from the bond connectivity and valence perspective but are not synthetically accessible, taking into account the current level of synthetic chemistry technologies (Fig. 1, I). These structures are obviously out of medicinal chemists’ interest, and the SA models and their thresholds play an important role as an *“event horizon”* to protect a medicinal chemist from the *“dark hole”* of synthetically inaccessible chemical space.

Compounding this is the non-uniform distribution of bioactivity within chemical space. Bioactive molecules tend to cluster in certain regions due to biological constraints, receptor compatibility, and synthetic accessibility. Consequently, naive random sampling or fragment-based combinatorial approaches often lead to low hit rates, while biased or knowledge-driven approaches (e.g., ligand-based design, scaffold hopping, generative models) are needed to navigate more promising subspaces.

Recent advances in generative chemistry and, particularly, AI-driven approaches such as deep generative models and reinforcement learning have significantly revitalized efforts to explore chemical space more effectively^7–12^. These methods are designed not only to sample chemical space more intelligently but also to optimize generated molecules with respect to multiple objectives, including potency, selectivity, and synthetic accessibility. However, systematic attempts to cover significantly larger portions of chemical space (e.g., >1 million structures) around a given drug target using modern AI-driven drug design (AIDD) and computer-aided drug design (CADD) techniques remain underreported. Such large-scale explorations are valuable not only for advancing our theoretical understanding of chemical space but also for practical purposes, such as identifying novel, patentable regions within it.

## Summary

In the current data descriptor, we are introducing the ultra-large-scale dataset of potential NLRP3 inhibitor molecular structures generated within the LEGION workflow, enabling massive coverage of the chemical space around the specified drug target. We have selected NLRP3 (NOD-, LRR- and pyrin domain-containing protein 3) for the study, since it is a high-value but complex drug target with strong links to inflammatory pathologies. Targeting it holds promise for treating diverse diseases driven by chronic inflammation, but drug discovery efforts must overcome selectivity, delivery, and mechanistic challenges. Several small-molecule inhibitors have been developed to target NLRP3^13–20^; however, none of them have reached the market, keeping the intrigue on the delivery of the first-in-class NLRP3 inhibitor.

As a result, LEGION successfully identified over 34,000 scaffolds aligned with 3D spatial binding hypotheses and subsequently generated approximately 110 million chemical structures. These resulting datasets of generated and enumerated structures provide an unprecedented opportunity to explore the capabilities of the 3D Generative Chemistry Workflow and the AI Screening supported by 2D Structural Enumeration. The provided code (see Code availability section) enables the generation of ~123B structures fully covering the capabilities of the LEGION workflow applied to the NLRP3 target.

The scaffolds derived from the generated structures include key pharmacophore functional groups essential for interacting with NLRP3, effectively occupying the binding pocket, and providing optimal vectors for chemical substitutions. This approach ensures that the generated compounds exhibit both binding relevance and structural validity, bridging the gap between 2D generative models and meaningful 3D chemical space exploration. The described methodology enables scaffold hopping with existing compound libraries as well as the generation of entirely new chemical cores. This flexibility facilitates the identification of diverse chemical matter relevant to the target of interest.

The ability to massively generate compounds around prioritized scaffolds offers practical advantages in intellectual property, such as providing a breadth of supporting examples for umbrella patent applications. The released datasets might be a good example for establishing a new way to present the intellectual property space in chemistry, generative models training and benchmarking, as well as to be a source for novel NLRP3 inhibitor chemotypes *in silico* identification.

The latter was confirmed by two studies: retrospective and prospective (see **Usage Notes** section). It should be noted that this opportunity cannot be provided by well-known large-scale datasets of molecular structures like Enamine REAL^21^, confirming the unique ability of datasets generated within LEGION methodology to cover the chemical space around the particular target. The results of the described studies let us encourage the prospective users of the LEGION NLRP3 datasets to utilize them in large-scale virtual screening studies (e.g., deep docking) for the identification of novel NLRP3 inhibitor chemotypes.

## Methods

### LEGION workflow

The LEGION workflow is powered by the Chemistry42 AIDD/CADD platform^22^. While the platform is designed to generate or screen a finite number of molecular structures, the task of massive chemical space coverage requires additional modules and custom workflow nodes beyond its default configuration. Thus, the **LEGION** (Latent Enumeration, Generation, Integration, Optimization and Navigation) workflow was developed to overcome the limitations of the common Generative Chemistry Workflow or AI-guided screening workflows, where the Generative Chemistry Workflow uses generative AI models to sample novel desired molecular structures and AI-guided screening uses AI methods to iteratively select only the most likely candidates from a large existing library.

Although the LEGION workflow relies extensively on the capabilities of the Chemistry42 platform, it serves as a general-purpose framework that combines any generative models and AI-based screening methods. While there are plenty of generative approaches one can pick, we used the standard set of models available in the Generative Chemistry Workflow, which included neural-based approaches such as language and 3D point cloud-based model nach0-pc^23^ and graph normalizing flow-based model MolGrow^24^, as well as a mix of evolutionary algorithms with a similar concept to JANUS^25^.

Under the AI screening methods umbrella, we mostly consider active learning^26^ and similarity-based^27^ methods, which still rely on some accurate computational feedback from simulation to be trained on, with AI scores being used to select the next promising candidates, hence AI-guidance. Moreover, we picked only one pretrained embedding similarity-method based on the reaction-aware representation^28^ that was more robust than a simple extended connectivity fingerprints-based similarity search, which starts with random clusters. More importantly, such embedding similarity-based methods are usually easily scaled to much larger datasets that we want to cover because they do not require expensive constant re-training compared to active learning methods.

It is worth noting that, compared to purely AI-only workflows that rely only on non-grounded AI scores, AI-guided Screening and the Generative Chemistry Workflow employ an additional reward feedback/filtering step for each AI-selected or AI-generated molecule based on accurate simulation and computational methods that are part of Chemistry42’s Reward component. We will cover which Reward components we picked in detail later on, but the framework the LEGION proposes is not restricted to the particular set of Reward functions and can be easily extended with, and especially for, computationally expensive physics-based methods that can handle only a handful of molecular structures in days.

**Fig. 2:**
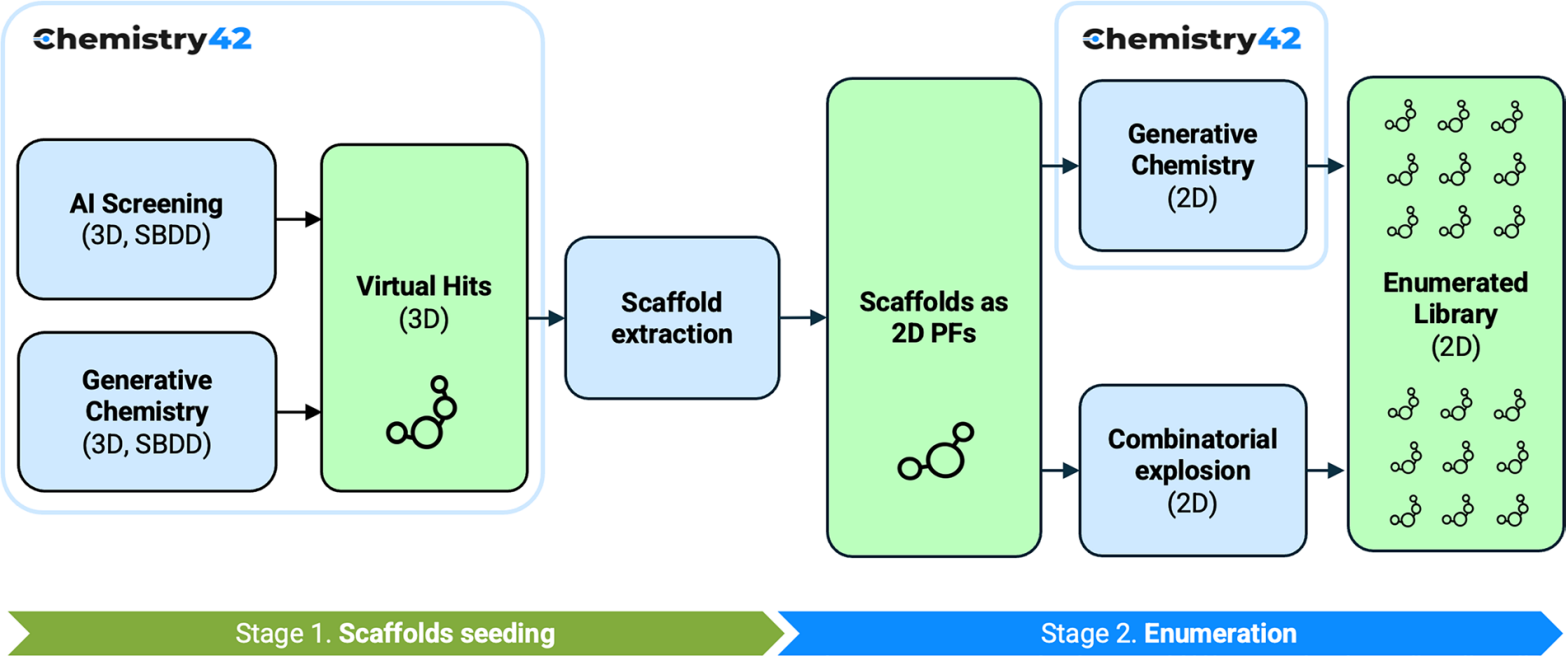
LEGION workflow. The blue boxes represent method nodes, and the green boxes represent molecular structure sets nodes.

The LEGION workflow starts from the experiments enabled by the Generative Chemistry Workflow and the AI Screening independently to collect as many scaffolds as possible (Fig. 2, Stage 1. Scaffold seeding). In-house (collected from scientific publications and patent applications), vendor (Enamine screening collection, libraries aggregated through MolPort), and public datasets (ChEMBL v34, PubChem) of synthetically feasible compounds are the input of AI Screening. Since the synthetic feasibility status of the molecules of such datasets is not questionable (Fig. 1, Fraction III), only Soft ReRSA (Retrosynthesis-Related Synthetic Accessibility)^29^ model policy and higher thresholds were applied during the AI Screening, as well as a minimal set of MCFs (Medicinal Chemistry Filters for structural alerts). In contrast, the Generative Chemistry Workflow employed a stricter ReRSA policy and a moderate set of MCFs to enable effective exploration of Fraction II of chemical space, which contains novel but not yet synthesized structures. Both AI Screening and Generative Chemistry utilize a 3D SBDD (Structure-Based Drug Design) workflow to identify and generate virtual hits, respectively, and use PDB files of co-crystallized ligands as a structural input for pharmacophore hypothesis modelling and scoring, ligand shape scoring, and docking.

After the 3D conformations of the docked virtual hits are collected from both the AI Screening and the Generative Chemistry Workflow, they are submitted to a scaffold extraction procedure, which is performed using an in-house 3D-pharmacophore-aware scaffold extraction algorithm. In this context, a **scaffold** is defined as *the 2D substructure of a 3D molecular conformation that is responsible for key protein-ligand interactions, as captured by one or more key 3D pharmacophore points*. These key 3D pharmacophore points must be user-defined, based on the structure-activity relationship (SAR) analysis of ligands targeting the protein of interest. For example, in the case of kinase inhibitors, such key pharmacophore points typically correspond to a hydrogen bond acceptor and donor pair located in the hinge region of the kinase orthosteric binding site. The developed algorithm is superior to the well-known, but ignorant of protein-ligand interaction, Bemis-Murcko scaffoldization^30^.

The extracted scaffolds are stored as SMILES strings, with asterisks (*) denoting attachment points. In this format, they can be reused as 2D privileged fragments within a 2D-tailored customization of Chemistry42’s Generative Chemistry Workflow, enabling rapid chemical space enumeration during **Stage 2** of the LEGION workflow. In addition to virtual scaffolds, we also collected corresponding peripheral fragments. Together with their peripheral fragments, these scaffolds can be independently used as input for simple combinatorial enumeration engines based on Cartesian product logic, facilitating unsophisticated but broad exploration of chemical space.

The enumerated library of 2D structures generated via both the 2D Generative Chemistry Workflow and Combinatorial explosion should be validated through 3D virtual screening experiments to estimate the probability that a given 2D structure can achieve the status of a 3D virtual hit. Without this justification, the enumeration of extremely large chemical subspaces offers limited practical value.

At **Stage 3** of the LEGION workflow, randomized subsets of the enumerated library are submitted to a 3D structure-based virtual screening (SBDD) workflow, mirroring the experimental setup used in **Stage 1**. The fraction of molecules that successfully pass the screening provides an **extrapolated probability estimate** for the **virtual hit rate** across the entire enumerated library.

### Ultra-large-scale dataset of potential NLRP3 inhibitors molecular structures

#### Input structural data

**Fig. 3:**
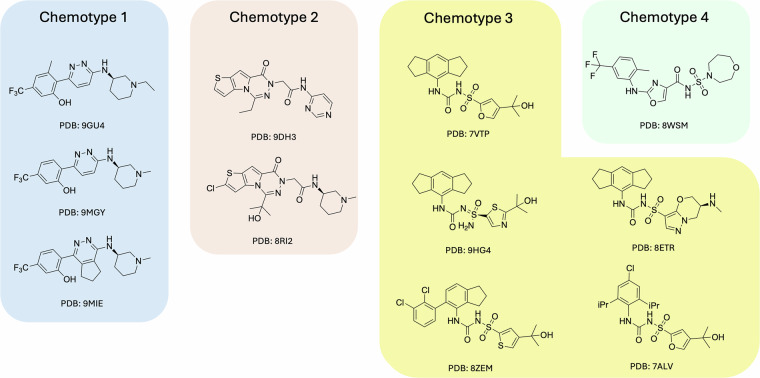
The chemotypes of co-crystallized NLRP3 inhibitors.

The small-molecule ligands reported as co-crystallized with NLRP3 in PDB were divided into 4 main chemotypes as described in Fig. 3.

**Fig. 4:**
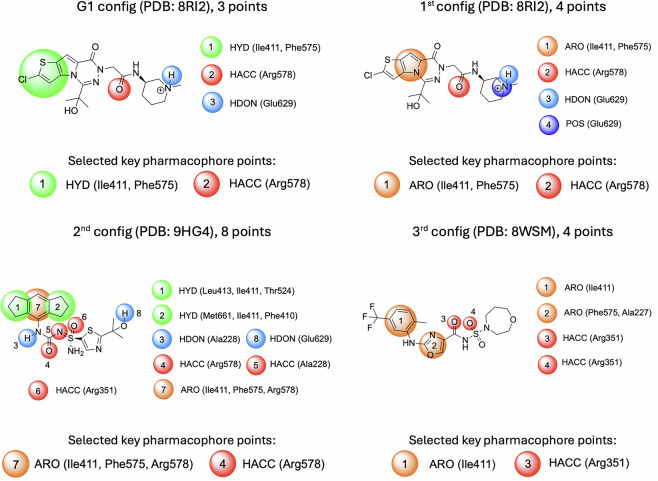
Ligand-based pharmacophore hypotheses designed for scaffold seeding experiments from co-crystallized NLRP3 inhibitors and key pharmacophore points selected for each experiment.

The most general pharmacophore was derived at Chemistry42 from the **NP3–562** ligand^15^, taken from PDB 8RI2. This hypothesis consists of 3 pharmacophore points: a HYD point in the hydrophobic subpocket (Ile411, Phe575), a hydrogen bond acceptor (Arg578), and a hydrogen bond donor (Glu629) (Fig. 4, G1 config). This general pharmacophore allows for satisfying most of the depicted ligands (Fig. 3).

The remaining three pharmacophore configurations (1^st^, 2^nd^, and 3^rd^) were designed to satisfy the SAR explanations from the publications related to the corresponding ligands^13,15,16^ (PDB codes: 8RI2, 9HG4, and 8WSM). **Chemotype 1** (Fig. 3) was not supported by a separate configuration since it was well fitted by the G1 and the 1^st^ pharmacophore configurations. This compatibility was found during the preliminary calibration screening studies for the ligands (Fig. 3) at the Chemistry42 platform.

Since the pharmacophore-aware scaffold extraction that we introduced for the LEGION workflow requires the definition of key pharmacophore points, we provided the following for each configuration (Fig. 4).

For the AI Screening experiments, we used the screening dataset of 15 M compounds collected from PubChem, ChEMBL, Enamine screening collection, MolPort, and scientific literature. The experiment configuration included Soft ReRSA policy, a higher ReRSA threshold (8.0), and the minimal set of MCFs. Shape scoring, Pharmacophore scoring, and docking-based PLI (Protein-ligand interaction) scoring were used as the main components of the Reward function and as the key drivers of AI-driven search of virtual hits. We carried out one AI Screening experiment for each SBDD Configuration (See Exp ID 1, 3, 5, and 7 in Table 1).

**Table 1:** The Stage 1 experiment statistics.

Configuration	Exp ID.	Experiment type	Reward configuration	Virtual hits	Crude scaffolds
**G1** (PDB: 8RI2)	1	AI Screening	Default	34,172	5,513
	2	Generative chemistry	CRV + SPAG	18,889	5,751
	X	Generative chemistry	Default	16,686	5,419
	Y	Generative chemistry	CRV	18,315	5,104
**1st** (PDB: 8RI2)	3	AI Screening	Default	36,732	4,523
	4	Generative chemistry	CRV + SPAG	21,105	3,367
**2nd** (PDB: 9HG4)	5	AI Screening	Default	11,658	350
	6	Generative chemistry	CRV + SPAG	16,686	558
**3rd** (PDB: 8WSM)	7	AI Screening	Default	12,991	693
	8	Generative chemistry	CRV + SPAG	21,488	2,848
**Total crude scaffolds**					34,126

The default Reward function configuration of the Chemistry 42 platform is designed to balance between (1) the optimization of the Reward function value for the most promising chemotypes, enlarging the size of the corresponding clusters, and (2) the discovery of new clusters. Technically, the **scaffolds** that we defined in this research paper are a good representation of the chemotypes and clusters of Chemistry42. Thus, for the experiments enabled by the Genereative Chemistry Workflow, we implemented two Reward function modifications to satisfy the ideology of the LEGION workflow to collect as many scaffolds as possible.

The first modification was dedicated to equalizing the Reward function values by a Constant Reward Value (CRV) for all generated structures that passed the filtering threshold for every pipeline module, in order to prevent the generative models from focusing on particular clusters for their further enlargement and optimization. The second implemented modification (SPAG, Similarity Penalty to Already Generated) aimed to penalize the generated structures if they are similar to the already generated ones. Both new modifications, if applied simultaneously (CRV + SPAG), positively affect the generations by an increased number of virtual hits (around 13%) and an increased number of scaffolds (See Exp ID 2, X and Y in Table 1). Thus, the CRV + SPAG reward configuration was applied to all remaining SBDD setups of the generative pipeline. The Generative Chemistry Workflow experiments configuration included a Strict ReRSA policy, a moderate ReRSA threshold (6.5), and three sets of MCFs (minimal, moderate, and covalent). Shape scoring, Pharmacophore scoring, and docking-based PLI (Protein-ligand interaction) scoring were used as the main components of the Reward function, while after passing the reward modules, filtering all reward values were capped to the constant value (CRV).

The customized BRICS algorithm^31^ with retrosynthetic intermediates kept during the BRICSDecompose was used to fragmentize the virtual hits. The **primary scaffold** for each molecule was extracted as the smallest BRICS fragment out of all fragmentation products of BRICSDecompose with at least one attachment point (*), having at least one cycle, and matching all key pharmacophore points. Then all **primary scaffolds** with 1–3 attachment points (*) are collected together, neutralized using RDKit^32^ neutralizer (Atom.SetFormalCharge(0) for charged atoms), and duplicates are dropped, as well as scaffolds with more than 3 attachment points. That results in 34,126 unique **crude scaffolds** collected from all 10 SBDD experiments (the **DA4** set, as described in the **Data Records** section).

#### LEGION Stage 2. Enumeration

**Fig. 5:**
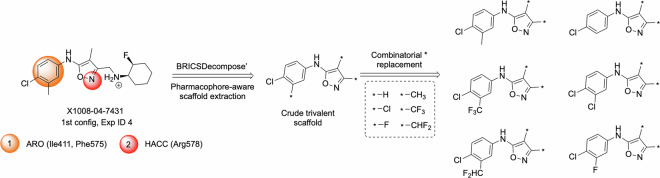
Combinatorial expansion of trivalent scaffolds to bivalent ones.

The module of 2D Privileged fragments at the Chemistry42 platform results in generating more candidates with the number of attachment points (*) per fragment up to 2, thus practically limiting to monovalent (one * in a structure) and bivalent (two * in a structure) scaffolds on realistic setups. That could mean that many scaffolds with 3 attachment points would be hard for generative models to generate, and reward functions would filter out many such generated attempts. To overcome this limitation, we have performed combinatorial replacement of one attachment point with the small fragments for such trivalent scaffolds. These replacements are applicable to the attachment points on aromatic carbons (see Fig. 5).

The set of **final scaffolds** derived after the described combinatorial replacements consists of 93,676 monovalent and bivalent scaffolds. These scaffolds were then used as 2D Privileged fragments (PFs) for the experiments within the 2D Generative Chemistry Workflow at the Chemistry42 platform. To ensure the maximal coverage of the scaffolds during the enumeration, the dataset was broken into 94 subsets of a maximum of 1000 PFs per experiment, thus 94 experiments were launched. The configuration of such experiments included Soft ReRSA policy and moderate ReRSA threshold (6.5) and three sets of MCFs (minimal, moderate, and covalent). No 3D modules (e.g., Pharmacophore, Shape, and Docking) were included at this Stage, since it would be computationally unfeasible.

**Fig. 6:**
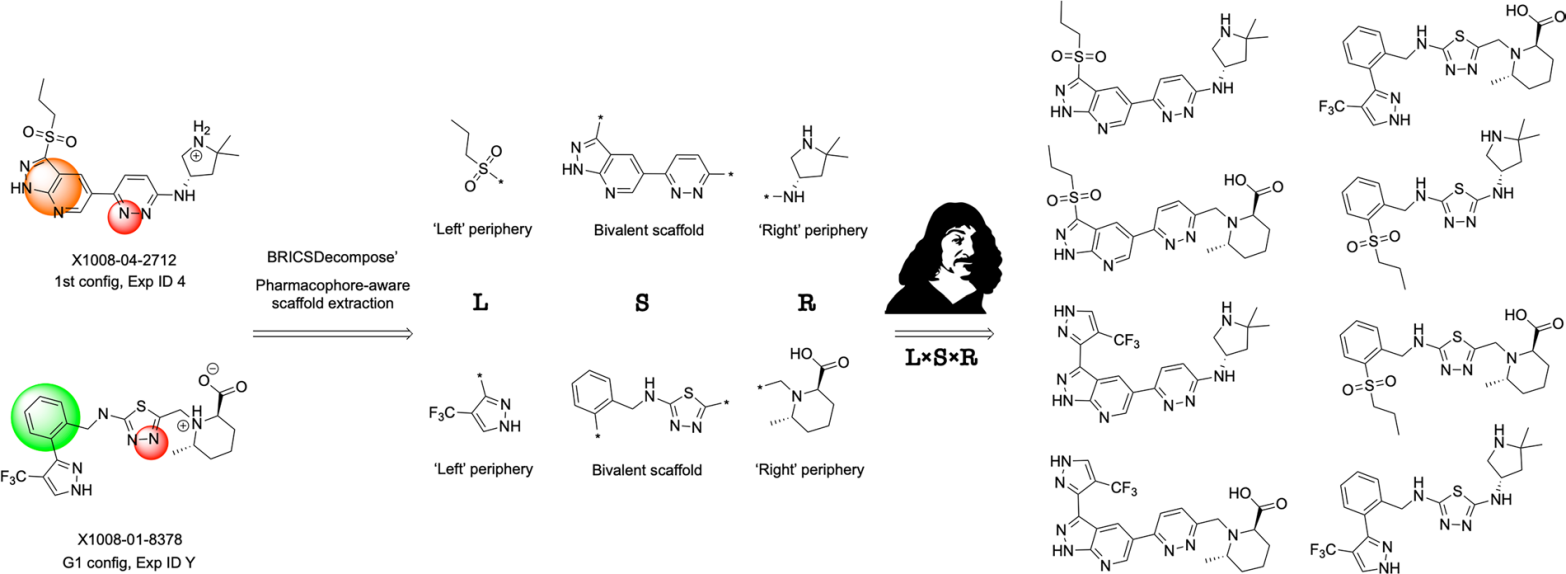
Peripheral fragments extraction example and Cartesian product of scaffolds by them.

In order to cover the chemical space more excessively via Combinatorial explosion following the logic of Cartesian product, we have designed a simple combinatorial generator that takes the bivalent crude scaffold from the virtual hits and multiplies it by the 2 sets of peripheral fragments (so-called ‘left’ and ‘right’ ones) extracted from the same virtual hits (see Fig. 6). The proposed classification of the ‘left’ and ‘right’ peripheral fragments is made arbitrarily and follows from the 3D orientation of a source molecular structures (co-crystals): the ‘left’ fragment corresponds to the hydrophobic subpocket, while the ‘right’ fragment corresponds to the polar subpocket of the NLRP3 binding site.

Finally, we prepares a subset of 11,935 unique bivalent scaffolds, 1,288 ‘left’ and 7,985 ‘right’ peripheral fragments applicable to the developed Combinatorial explosion algorithm. The complete Cartesian product of these items gives ~123B of structures. However, due to the technical limitations here, we are providing a portion of this chemical space (see DA6 in the Data Recors section). Thus, random sampling of 100 ‘left’ and 100 ‘right’ fragments per each scaffold gave us a reduced and technically feasible number of ~119 M molecular structures, which then were submitted to MCFs filtering (minimal, moderate, and covalent subsets). At the same time, in order not to limit the capabilities of the workflow and to allow for ultimate navigation of the ~123B structures we are providing the full list of the above-mentioned scaffolds and fragments alongside with the code enabling full-scale combinatorial enumeration (see Code Availability section) to obtain these ~123B structures.

The efforts from the 2D Generative Chemistry Workflow and Combinatorial explosion resulted in the enumeration of ~6.5 M and 100 M 2D molecular structures, respectively. Both datasets are provided with this article and are referred to as the **DA5** and **DA6** sets in the Data Records section.

## Data Records

The datasets referenced in the **Method** section can be accessed on Zenodo^33^. Molecular data are stored either in SDF (Structure Data File) format, where 3D poses are provided, or in CSV (Comma Separated Values) format when only SMILES notation is included. A brief summary of each file’s contents is provided below.

Table 2 lists the available fields for the following SDF files:

**Table 2:** Content types for the SDF files (**DA1,** **DA2,** **DA7***–***DA9**).

Property name	Description
Structure ID	Serial number of the compound from the initial input dataset for the AI screening
Matched Pharmacophore Points	Default fields available for the screened / generated molecular structures on the Chemistry42 platform (see ref. ^22^)
Pharmacophore Score	
PLI Score	
Shape Score	
ReRSA	
Rewarded Isomeric SMILES	Compound structure in SMILES notation
exp_id or Experiment_config	Experiment ID or configuration consistent with Table 1

**DA1** *. AI Screening hits, 3D poses (~95 K), SDF*

**DA2** *. 3D Generative Chemistry Workflow hits, 3D poses (~113 K), SDF*

**DA7** *. 2D Generative Chemistry Workflow output sample re-screened, hits, 3D poses (220 K), SDF*

**DA8** *. Combinatorial explosion output sample re-screened, hits, 3D poses (29 K), SDF*

**DA9** *. GenGramm exploration + MolGrow exploitation setup hits, 3D poses (9 K), SDF*

The poses from the SDF files above can be visualized in the pocket environments. The preprocessed co-crystal structures are accessible as PDB files at **DA3**. These PDB files (three in total, with names matching those in Table 1) can be used to view the 3D poses.

Extracted crude scaffold structures are available in **DA4**. ~34 K records are included, each linked to the source molecular structure. The fields available are listed in Table 3.

**Table 3:** Content types for the **DA4** CSV file.

Column name	Description
molecule_id	ID of the molecular structure, the scaffold was extracted of
smiles	Molecular structure in SMILES notation, the scaffold was extracted of
scaffold	The structure of scaffold in SMILES notation
experiment_id	The experiment ID where the molecular structure was obtained

The CSV file for the 2D Generative Chemistry Workflow output contains ~6.5 M records, with data organized in SMILES notation. It includes calculated 2D descriptors such as ReRSA, MCE-18^34^, MW, HBA, HBD, SLogP, and TopoPSA (refer to **DA5***. 2D Generative Chemistry Workflow output*).

For the Combinatorial explosion output, 100 M records are provided in the CSV file with structures in SMILES notation (see **DA6***. Combinatorial explosion output*). This CSV file contains only one column with SMILES strings with no other descriptors added.

## Technical Validation

**Fig. 7:**
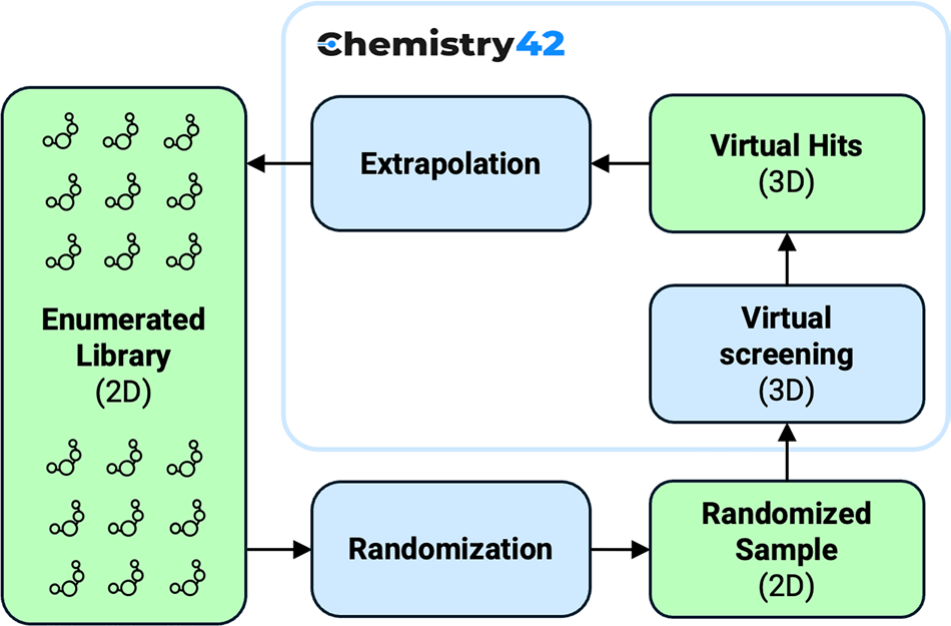
Technical validation workflow for 2D enumerated part of the NLRP3 dataset.

To ensure that the chemical space fraction generated at Stage 2 makes sense from the 3D SBDD perspective, we have performed an extrapolation study within random sampling from the enumerated library and subsequent 3D SBDD Virtual Screening at the Chemistry42 platform, mirroring the experimental setup used in **Stage 1 (see** Fig. 7**)**. The fraction of molecules that successfully pass the screening provides an **extrapolated probability estimate** for the **virtual hit rate** across the entire enumerated library.

To evaluate the extrapolation power of the 2D Generative Chemistry Workflow, we took a sample of 375,000 molecular structures and assessed them in G1 SBDD configuration (see Table 4). Also, 4 random samples of 50,000 molecular structures were taken from the Combinatorial explosion part of the Enumerated library and then subsequently evaluated in all 4 SBDD configurations (G1, 1^st^, 2^nd^ and 3^rd^). Soft ReRSA policy and moderate ReRSA threshold (6.5), filtering by three sets of MCFs and key 3D modules (Pharmacophore, Shape, and Docking) were enabled for all 3D SBDD Virtual Screening experiments at this Stage. Finally, the 2D Generative Chemistry Workflow allowed for more than 60% extrapolation from 2D structures to 3D virtual hits, while the 3D virtual hit-rate for structures generated by the combinatorial approach fluctuated between 8 and 26% depending on the SBDD experiment configuration. Both rescreened datasets with 3D poses of structures passed all reward components of the respective SBDD configurations (**DA7** and **DA8**, as described in the Data Availability section) are provided.

**Table 4:** Extrapolation study of latent 2D subspace to 3D virtual hits.

Sample source	Sample size	Configuration	3D Virtual hits	3D Virtual hit rate
2D Generative chemistry	375,000	G1	220,466	58.8%
Combinatorial explosion	50,000	G1	13,090	26.18%
Combinatorial explosion	50,000	1^st^	4,457	8.91%
Combinatorial explosion	50,000	2^nd^	6,821	13.64%
Combinatorial explosion	50,000	3^rd^	5,119	10.24%

## Usage Notes

The users of the LEGION NLRP3 datasets are encouraged to utilize them for large-scale virtual screening campaigns to identify novel and diverse chemotypes of NLRP3 inhibitors. The proposed usage is justified within two studies: retrospective and prospective.

### Retrospective study on the dataset usage

It was mentioned that we had not designed a separate pharmacophore hypothesis for **Chemotype 1** (Fig. 3), since it is well-fitted by both G1 and 1^st^ configurations. However, the very fact of the pharmacophore similarity between Chemotype 1 and Chemotype 2 is not trivial and was revealed by the respective study by Novartis^14^. Thus, we were wondering whether it is possible to find the **Chemotype 1** scaffold using AI Screening without direct injection of the chemotype knowledge into the input configuration.

**Fig. 8:**
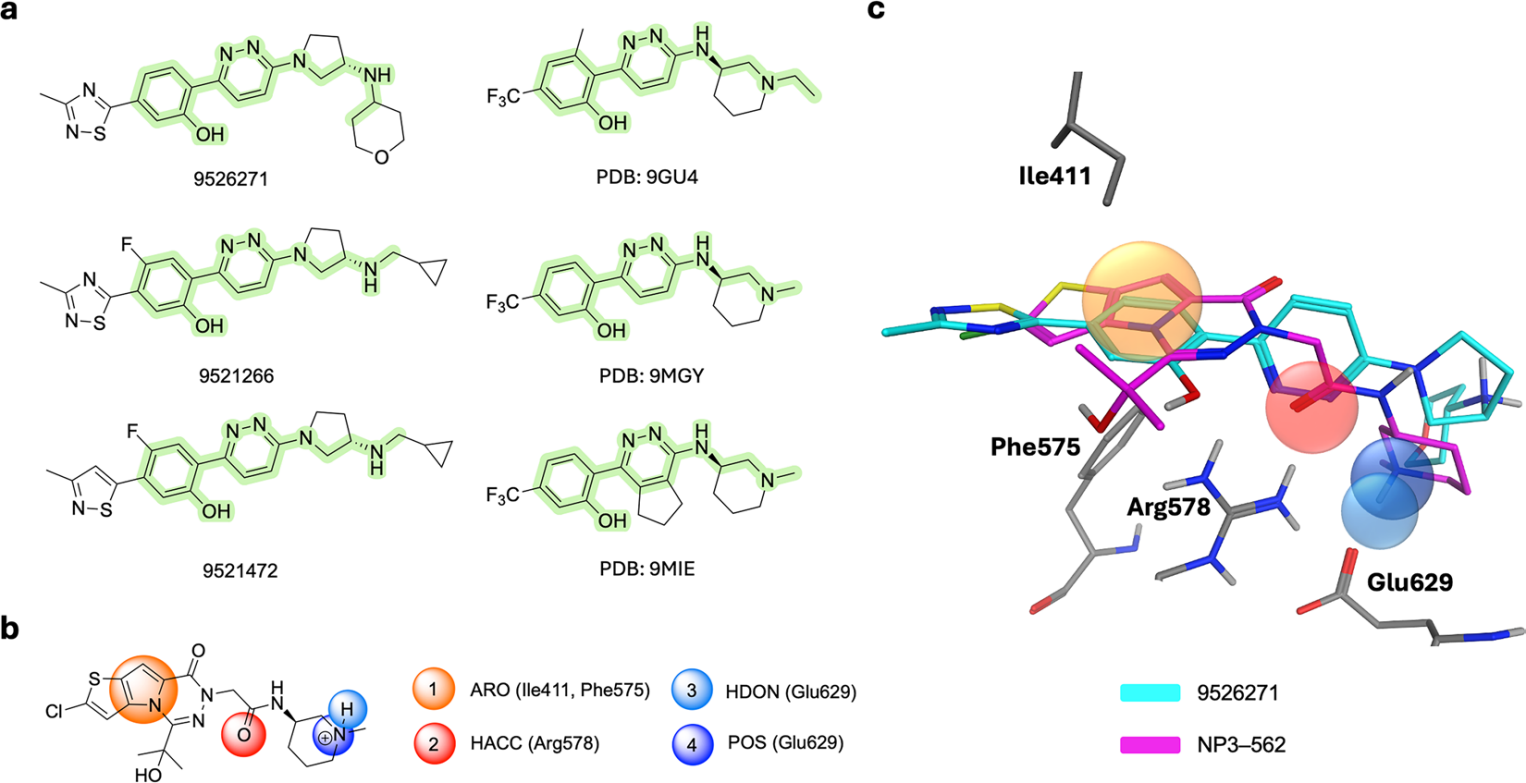
**Chemotype 1** scaffold found from an NLRP3 unrelated source during AI screening. (**a**) MCS is highlighted (green) for virtual hits and **Chemotype 1** ligands, (**b**) 2D depiction of the pharmacophore hypothesis derived from PDB 8RI2 ligand utilized in the 1^st^ config, (**c**) Alignment of **Structure ID 9526271** (cyan), (see **DA1**) of compound derived from patent WO2023034836A1 and PDB 8RI2 ligand (NP3–562, magenta), (see **DA3**).

Interestingly, we were able to find such examples, where the **Chemotype 1** scaffold was present in the identified virtual hits. They share a large maximum common substructure (MCS)^35^ with **Chemotype 1** examples (Fig. 8a). What is even more interesting is that these molecules are sourced from the WO2023034836A1^36^ patent application by Remix Therapeutics Inc., while the target claim for compounds exemplified in this patent application is distinct from NLRP3. When aligned together (see Figs. 8b and 8c) with compound **NP3–562** in 3D-frame of PDB: 8RI2 (see **DA3** in Data availability section), the found virtual scaffold matches the structural features of **Chemotype 1** examples in the same way as it was shown for compound **NP3-253** (a brain-penetrant representative of **Chemotype 1**, which is actually a ligand from PDB: 9GU4) in the respective article by Novartis.

**Fig. 9:**
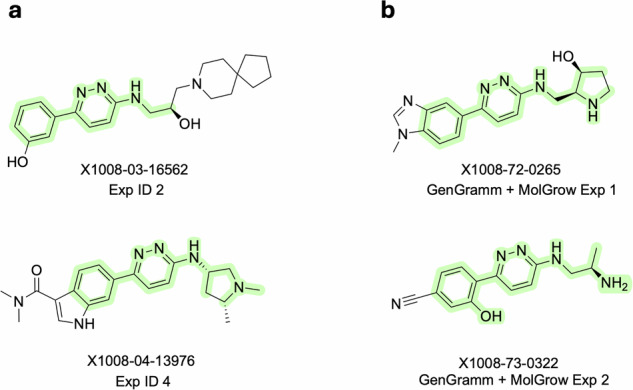
**Chemotype 1** scaffold found from an NLRP3 unrelated source during the experiments within the Generative Chemistry Workflow. (**a**) **Chemotype 1** representatives generated during Exp IDs 2 and 4 by the ensemble of generative models. MCS is highlighted (green) for virtual hits and **Chemotype 1** ligands. (**b**) **Chemotype 1** representatives generated by 2 generative models (see **DA9**) in 2 separate experiments (1 and 2). MCS is highlighted (green) for virtual hits and **Chemotype 1** ligands.

The demonstrated example of *in silico* scaffold-hopping from **Chemotype 2** to **Chemotype 1** scaffolds was enabled within both the AI Screening (Fig. 8) and the Generative Chemistry Workflow (see Fig. 9).

These findings even mean that we could potentially identify **Chemotype 1** using the Generative Chemistry Workflow, right after getting the access to the ligands of **Chemotype 2** (Fig. 3), which are precedent to **Chemotype 1**, as claimed in the paper by Novartis (Fig. 9b) and clear from the corresponding patent applications by Novartis for **Chemotype 2** (WO2020021447A1^37^, filing date: July 23th, 2019) and for **Chemotype 1** (US11208399B2^38^, filing date: May 15th, 2020). Of course, it is hard to say whether **Chemotype 1** would be chosen for further development as part of the output of a Generative Chemistry Workflow experiment, if found *de novo* without any *posteriori* conclusions based on the data from the HTS. However, the very existence of the independent chemotype in the provided dataset makes it valuable from the view of prospective virtual screening studies for this large-scale dataset. Despite medicinal chemists prefer looking at *in vitro* data before nominating a new chemotype, the very fact that the proposed datasets could independently help to ideate some established chemotypes much faster than the temporal gap between patent applications of preceding and post-going chemotypes provides us with an understanding of its substantial capabilities for novel small molecule design.

### Prospective study on the dataset usage

The prospective validation study extremely highlights the potential of the proposed datasets usage, when the chemotypes that can be found in our dataset are reported as novel, diverse and active chemotypes by the independent researchers, far after the publication of our dataset.

**Fig. 10:**
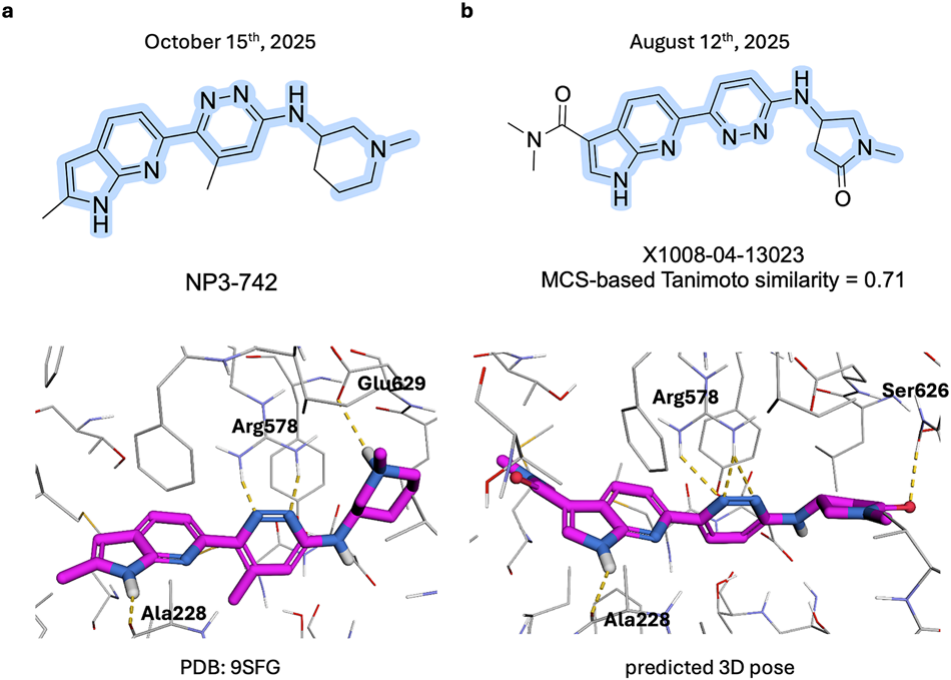
Comparison of 2D structures of NP3-742 (**a**) and X1008-04-13023 (**b**) (common substructure is highlighted in blue) and their 3D poses (crystallographic in case of NP3-742 (**a**) and predicted by Chemistry42 in case of X1008-04-13023 (**b**)).

Such a novel, diverse and active chemotype was reported on October 15^th^ by the Novartis team in a respective article in the Journal of Medicinal Chemistry^39^. The unusual replacement made for the phenol moiety, converted into azaindole, made it possible for the Novartis team to nominate the novel chemotype of the NLRP3 inhibitors exemplified by the compound NP3-742. This chemotype cannot yet be detected (as of November 21^st^, 2025) within the patent application search conducted via SciFinder^40^, either because the respective patent application has not yet been published online or it has not yet been indexed within SciFinder. The molecular structures (more than 4200) sharing the large MCS with the novel Chemotype were surprisingly found in the LEGION NLRP3 potential inhibitors datasets (DA2 and DA6) deposited on Zenodo^33^ on August 12^th^. The provided 3D structure (X1008-04-13023, which can be found in DA2, see Data Records) of the shared chemotype also fits the newly reported binding pose of the chemotype (PDB: 9SFG, published at RSCB on 22^nd^ October; see Fig. 10).

It should be noted that this finding within the NP3-742 chemotype substructure (see SI1) cannot be reproduced within well-known large-scale datasets of virtual and synthetically accessible structures, such as Enamine REAL^21^. The search in these datasets (Table 5) enabled within DataWarrior^41^ does not result in any matches. This highlights the extreme importance of the target-oriented large-scale datasets of potentially active molecular structures, rather than the usage of large, but still sparse, chemical spaces provided by vendors.

**Table 5:** The substructure search was made using Alipheron’s Hyperspace Search^42^ implemented in DataWarrior.

Chemical space name	Size
ChemSpace Freedom Space	142 B
Enamine REAL Space	83 B
LifeChemical LifeCheMyriads	26.7 B
PharmaBlock Sky Space	56.8 B
WuXi AppTec GalaXi	28.6 B
eMolecules eXplore-Synple	5.32 T
Molecule.one D2B-SpaceM1	1.49 B

### Supplementary materials

The scaffold-defining substructure of the novel and diverse NLRP3 inhibitor chemotype, exemplified by NP3-742, used for searching large-scale datasets using Alipheron’s Hyperspace Search implemented in DataWarrior, is provided in SI1.

The limitations and the applicability domain of the LEGION workflow to produce the large-scale target-oriented dataset, such as presented in the current paper, are provided in the supplementary materials (see SI2).

## Supplementary information

Supplementary Information

## Data Availability

At present, the following datasets are available at Zenodo^33^: DA1. AI Screening hits, 3D poses (~95 K), SDF DA2. 3D Generative Chemistry Workflow hits, 3D poses (~113 K), SDF DA3. PDB frames to view 3D poses (3 files), PDB DA4. 2D crude scaffolds derived from 3D virtual hits (~34 K), CSV DA5. 2D Generative Chemistry Workflow output (~6.5 M), CSV DA6. Combinatorial explosion sample output (100 M), CSV DA7. 2D Generative Chemistry Workflow output sample re-screened, 3D poses (220 K), SDF DA8. Combinatorial explosion output sample re-screened, 3D poses (29 K), SDF DA9. GenGramm exploration + MolGrow exploitation setup hits, 3D poses (9 K), SDF See detailed description in the **Data Records** section.

## Code availability

Chemistry42 is an industry-grade commercial software platform available at http://chemistry42.com.

The code enabling combinatorial explosion and the ultimate coverage of 123B chemical space is available on GitHub: https://github.com/insilicomedicine/legion-scaffold-decorator. The lists of SMILES strings of bivalent scaffolds (11,935), ‘left’ (1,288) and ‘right’ (7,985) peripheral fragments enabling the full coverage of combinatorial space (~123B) are also provided.
