# Supplementary material for: Molecular LEGION: incalculably large coverage of chemical space around the NLRP3 target
Source: Sci Data. 2026 Mar 3;13:576. doi: 10.1038/s41597-026-06850-y (PMC13065923; doi:10.1038/s41597-026-06850-y)
Supplement: Supplementary file 1 — Supplementary Information [file 41597_2026_6850_MOESM1_ESM.pdf]

Molecular LEGION: incalculably large coverage of chemical space around the NLRP3 target

Bogdan Zagribelnyy[a], Vladimir Aladinskiy*[a], Nikita Bondarev[a], Ivan Ilin[a], Maxim Malkov[a], Anna Vasileva[b], Xiaoyu Ding[c], Arkadii Lin[a], Rim Shayakhmetov[a], Alex Aliper[a,b], Feng Ren[c], Alex Zhavoronkov[a,b,d]

[a] *Insilico Medicine AI Limited, Level 6, Unit 08, Block A, IRENA HQ Building, Masdar City, Abu Dhabi, UAE*
[b]*Insilico Medicine Hong Kong Ltd., Unit 310, 3/F, Building 8W, Phase 2, Hong Kong Science Park, Pak Shek Kok, New Territories, Hong Kong, Hong Kong SAR, China*
[c]*Insilico Medicine Shanghai Ltd., Suite 901, Tower C, Changtai Plaza, 2889 Jinke Road, Pudong New District, Shanghai 201203, China*
[d]*Insilico Medicine Canada Inc., 3710-1250 René-Lévesque Blvd W, Montreal, Quebec, H3B 4W8, Canada*

* *Corresponding Author Email: vladimir@insilicomedicine.com*

Supplementary Information

SI1. Alipheron's Hyperspace Search configuration within DataWarrior.

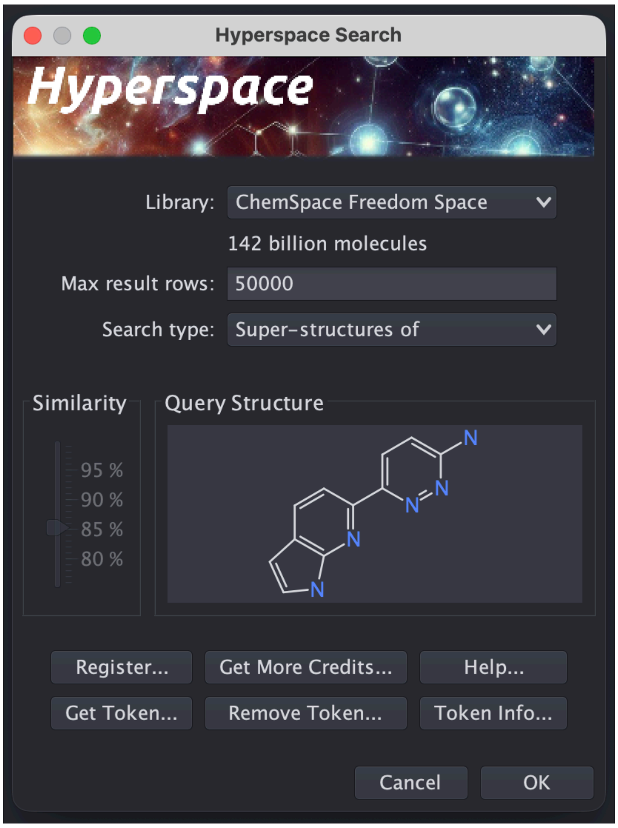

The substructure of the NLRP3 inhibitor chemotype, exemplified by NP3-742 and used as a query in Alipheron's Hyperspace Search, is shown in the Query Structure field.

SI2. Limitations and Applicability Domain

The size of the final chemical space coverage heavily depends on the availability of data related to the target of interest and, consequently, the number of experimental configurations that can be created. In the generation of potential NLRP3 inhibitors dataset, we utilized published co-crystal structures and SAR data to design four distinct configurations. With a sufficient amount of target information, we successfully generated tens of millions of structures, with the potential to reach billion-scale using the combinatorial engine. However, data availability is specific to each target, and smaller-scale chemical space coverage is expected for cases lacking 3D structural information or SAR data.

The first stage of the workflow combines the AI Screening and the Generative Chemistry Workflow, both components of the Chemistry42 platform, which operate within a paradigm of flexible input data. Both methods can function in SBDD or ligand-based drug design (LBDD) modes, depending on whether the input consists of a protein-ligand complex or a ligand structure alone. Importantly, the success of pharmacophore-aware scaffold extraction depends on providing meaningful pharmacophore hypotheses. In an LBDD scenario, the best practice involves deriving pharmacophore hypotheses from reliable SAR data to enhance the relevance of the extracted structural cores and their target interactions. Conversely, in an SBDD approach, pharmacophore hypotheses can be directly derived from protein-ligand interactions in the co-structure, assuming the experimental or modeled structure is of sufficient quality.

Pharmacophore scoring is also critical at the **Technical validation** stage, particularly for assessing the reliability of 2D-enumerated structures derived from LBDD experiments conducted at **Stage 1**. Insufficient SAR information early in the process can lead to the generation of less relevant chemical space coverage in subsequent stages.

During **Stage 2**, the workflow introduces two distinct methods for library enumeration, each with its respective advantages and limitations. The 2D Generative Chemistry Workflow can produce hundreds of thousands of compounds within 1-2 days, while the Combinatorial Explosion engine is capable of generating tens or even hundreds of millions of structures. However, follow-up technical validation within 3D virtual screening from the potential NLRP3 inhibitors dataset revealed that approximately 60% of the 2D structures generated using Chemistry42 aligned with 3D binding hypotheses. In contrast, only 8-26% of the structures output by the combinatorial engine satisfied the same criteria. This highlights a trade-off between the scale of enumeration and the structural relevance of the resulting library.
